# Aircraft Pose Estimation Based on Geometry Structure Features and Line Correspondences

**DOI:** 10.3390/s19092165

**Published:** 2019-05-09

**Authors:** Xichao Teng, Qifeng Yu, Jing Luo, Gang Wang, Xiaohu Zhang

**Affiliations:** 1 College of Aerospace Science and Engineering, National University of Defense Technology, Changsha 410073, China; tengari@buaa.edu.cn (X.T.); yuqifeng@vip.sina.com (Q.Y.); wanggang13@nudt.edu.cn (G.W.); 2 Qing Zhou High-Tech Institute, Weifang 262500, China; luoj11@tsinghua.org.cn; 3 School of Aeronautics and Astronautics, Sun Yat-Sen University, Guangzhou 510000, China

**Keywords:** aircraft pose estimation, wide-baseline image pairs, structure extraction, bilateral symmetry, weak perspective projection, vector analysis, line correspondences

## Abstract

A robust and accurate aircraft pose estimation method is proposed in this paper. The aircraft pose reflects the flight status of the aircraft and accurate pose measurement is of great importance in many aerospace applications. This work aims to establish a universal framework to estimate the aircraft pose based on generic geometry structure features. In our method, line features are extracted to describe the structure of an aircraft in single images and the generic geometry features are exploited to form line groups for aircraft structure recognition. Parallel line clustering is utilized to detect the fuselage reference line and bilateral symmetry property of aircraft provides an important constraint for the extraction of wing edge lines under weak perspective projection. After identifying the main structure of the aircraft, a planes intersection method is used to obtain the 3D pose parameters based on the established line correspondences. Our proposed method can increase the measuring range of binocular vision sensors and has the advantage of not relying on 3D models, cooperative marks or other feature datasets. Experimental results show that our method can obtain reliable and accurate pose information of different types of aircraft.

## 1. Introduction

Aircraft pose estimation is a necessary technology in the field of aeronautical engineering and accurate pose parameters of the aircraft provide the fundamental information needed in many aerospace tasks, such as flight control system testing [1,2,3], auxiliary taking-off and landing [4], collision avoidance and autonomous navigation [5]. Various sensors are used to determine the position and attitude of aircraft, for instance, GPS sensors, altimeters and inertial measurement units (IMU) [6]. During the past decade, considerable progress has been made in visual sensors and computer vision technologies and a variety of vision-based methods have been developed for estimating the pose of a target. Compared to systems using GPS and IMU, vision systems are relatively inexpensive and can perform rapid pose estimation with the advantage of low power consumption, high flexibility and accuracy [7,8]. In recent years, performing robust and accurate aircraft pose estimation based on visual sensors has been an attractive research topic and remains quite challenging.

In general, the visual sensors used for aircraft pose estimation include RGB-D (Red Green Blue-Depth) sensors, binocular vision sensors and monocular cameras. RGB-D sensors provide RGB images and depth information [9,10] for pose estimation, binocular vision sensors estimate the pose of a target by extracting 3D information from two images containing overlapping regions [11,12,13], while monocular camera systems use single 2D images to estimate the pose information [14,15,16,17,18]. RGB-D sensors are referred to as active sensors while binocular vision sensors and monocular cameras are passive sensors.

The RGB-D sensor utilizes an active light source for the close-range sensing of 3D environments. Among visual based pose estimation methods, a simultaneous localization and mapping (SLAM) technique is often used to process the obtained image sequences efficiently [19,20,21,22]. RGB-D SLAM integrates visual and depth data for real-time pose estimation. References [23,24,25,26,27] presented on-board systems for real-time pose estimation and mapping using RGB-D sensors, which enabled autonomous flight of an unmanned aerial vehicle (UAV) in GPS-denied indoor environments with no need of wireless communication. The accurate and computationally inexpensive RGB-D SLAM is performed based on visual odometry, loop closure and pose graph optimization. To reduce the computational cost of RGB-D SLAM further, methods based on signed distance functions (SDF) were proposed in [28,29] for real-time UAV pose estimation. SDFs utilizes the distances to the surface of a 3D model to represents the scene geometry, and the pose parameters of an UAV are estimated by minimizing the error of the depth images on SDF. By using SDF, these methods yield comparable accurate and robust pose estimation to RGB-D SLAM at a much higher speed. To increase the accuracy of 3D rigid pose estimation for long term, Gedik and Alatan [30] fused visual and depth data in a probabilistic manner based on extended Kalman filter (EKF). Liu et al. [31] used multiple RGB-D cameras to estimate the real-time pose of a free flight aircraft in a complex wind tunnel environment. A cross-field of view real-time pose estimation system was established to acquire the 3D sparse points and the accurate pose of the aircraft simultaneously. Although the RGB-D sensor can obtain pose parameters with high precision, the short measuring distances limit its application to aircraft pose estimation in outdoor environments.

Binocular vision systems estimate the pose of the target through feature correspondence and 3D structure computation [32,33,34], and the depth information is usually extracted by stereo matching [35]. To estimate the aircraft absolute pose, 3D models or cooperative marks are also needed [36]. An on-board binocular vision-based system presented in [37] estimated the relative pose between two drones to verify the autonomous aerial refueling of UAVs. Xu et al. [38] proposed a real-time stereo vision-based pose estimation system for an unmanned helicopter landing on a moving target. The geometry features of 2D planer targets are used to simplify the process of feature extraction. The authors in [39] used a stereo vision SLAM to acquire accurate aircraft pose parameters for industrial inspection. This stereo vision SLAM estimates aircraft pose using stereo odometry based on feature tracking (SOFT) and a feature-based pose graph SLAM solution (SOFT-SLAM) is proposed. Many on-board stereo vision-based pose estimation algorithms are mainly developed for applications in which aircraft are close to 3D environments, i.e., the ratio of the stereo camera’s baseline to depth of field is relatively large. For an aircraft flying at high altitudes, it is hard to obtain accurate and robust depth information from stereo image pairs with a small baseline, and aircraft jitter also affects the stereo camera calibration. The baseline length of two cameras is an important parameter which affect the corresponding disparity between two images. A large baseline can improve the accuracy of depth estimation and increase the measurement range [40]. But with increasing the baseline distance, stereo matching becomes a quite challenging task for traditional binocular vision methods. The authors in [41] proposed a hybrid stereovision method to estimate the motion of UAVs. The hybrid stereovision system consisted of a fisheye and a perspective camera and no feature matching between different views is performed which is different from classical binocular vision methods. The method first estimates the aircraft’s altitude by a plane-sweeping algorithm; then, the fisheye camera is used to calculate the attitude, and the scale of the translation is obtained by the perspective camera; finally, these pose parameters are combined to acquire the UAV motion robustly.

Compared to the state-of-the-art pose estimation systems based on RGB-D and binocular vision sensors, the monocular vision system is relative light in weight, computationally less expensive, and is also suitable for long-range pose estimation. For an aircraft with an on-board monocular camera, its pose can be determined by sensing the 3D environments. An on-board monocular vision system based on cooperative target detection was established in [42] for aircraft pose estimation. Coplanar rectangular feature on a known checkerboard target is extracted to determine the pose parameters and a continuous frame detection technique is presented to avoid corners’ confusion. The authors in [43] presented a simple monocular pose estimation strategy for UAV navigation in GNSS-denied environments. Known 3D geometry features in observed scenes are exploited and a perspective-and-point (PnP) algorithm is selected to estimate the pose accurately. Benini et al. [44] proposed an aircraft pose estimation system based on the detection of a known marker. The on-board system performs high-frequency pose estimation for autonomous landing using parallel image processing. For environments without known prior information, monocular SLAM [45,46,47,48,49] or the structure from motion (SfM) [50,51] method can be applied to estimate the relative pose of aircraft in real-time.

However, for external monocular vision systems which estimate an aircraft’s absolute pose using its 2D projected images, these on-board vision techniques are not applicable because of the unpredictable motion of aircraft and unknown scale factor. It is quite challenging to obtain accurate pose information from a single 2D image of an aircraft, especially for long distance measurements where the size of the aircraft is relatively small compared to its average distance to the monocular camera.

Prior knowledge about an aircraft’s structure, such as 3D models or synthetic image datasets, is usually needed to facilitate the estimation of aircraft pose parameters. For monocular model-based methods, the pose parameters are calculated by minimizing the distance between corresponding features extracted from 2D images and the 3D model. The authors in [52] exploited 3D shape models to estimate the motion of targets in an unsupervised manner. In this method, a set of feature point trajectories are detected in images and pose estimation is performed by aligning the trajectories and the 3D model. To reduce problem dimension, subspace clustering is used to select visible part of targets’ convex hull as candidate matching points, and a guided sampling procedure is performed to obtain the alignment (i.e., the motion matrix). Reference [53] presented a monocular vision system located on a ship’s deck to estimate the UAV pose for autonomous landing. The 3D CAD (Computer Aided Design) model of the UAV is integrated into a particle filtering framework and the estimation results of the UAV pose are obtained and optimized based on the likelihood metrics.

To reduce the complexity of model-based pose estimation, features describing the aircraft’s structure are proposed and 3D pose is estimated by pattern matching. Reference [54] extracted rotation invariant moments to describe the aircraft silhouette from monocular image data and converted the detected features to an estimated pose through a nearest neighbor search algorithm. Breuers and Reus [55] used normalized Fourier descriptors for structure extraction and estimated the aircraft’s pose by searching the best match in a reference database. An aircraft pose recognition algorithm was proposed in [56] based on locally linear embedding (LLE) which reduces the dimensionality of the problem. LLE is employed to extract structural features as inputs of a back propagation neural network and the aircraft’s pose is obtained by searching for local neighbors. Reference [57] utilized contour features to describe the aircraft’s structure and estimate the relative pose information. The algorithm first projects a 3D model into 2D images in different views and establishes a contour model dataset; then, the invariant moment and shape context are used for contour matching to recognize the aircraft’s pose. Wang et al. [58] used central moment features to acquire the pose information of commercial aircraft in a runway end safety area. Based on the structure information extracted by the central moments and random sample consensus (RANSAC) algorithm, a two-step feature matching strategy is proposed to identify an aircraft’s pose.

Although a lot of features-based methods have been presented to reduce the complexity of model-based pose estimation, 3D models are still required to obtain high-quality synthetic aircraft image datasets which is necessary for reliable feature matching and accurate pose estimation. The usage of detailed 3D models or feature matching is storage- and time-consuming, and for different types of aircraft, it is necessary to prepare corresponding 3D models and/or feature datasets which also reduces the flexibility and efficiency.

In this paper, a vision system using two monocular cameras with a large baseline is presented for pose estimation of model-unknown aircraft. In practical applications, our vision system uses two cameras located on the ground to track a free flight aircraft simultaneously and estimate its real-time 3D pose. For every captured image, the camera orientation parameters are recorded and known. Compared to other vision methods based on detailed 3D models or feature datasets, our approach only utilizes a pair of 2D images captured at the same time to estimate the 3D pose of an aircraft robustly and accurately. Some prior assumptions about the general geometry structure of aircraft are exploited to recognize aircraft’s main structure and obtain 3D/2D feature line correspondences for pose solutions. With a very large baseline distance, our system can perform long range measurement and enhance the accuracy of pose computation. By utilizing common structure features of aircraft, the line correspondences are established without relying on 3D models and there is no need for stereo matching which is very difficult for wide-baseline images.

Compared with other systems using binocular stereo vision sensors for aircraft pose estimation, our vision system enables binocular vision sensors to achieve a larger measuring range by adopting a much wider baseline configuration, and our method can estimate the aircraft pose from the wide-baseline image pairs robustly and accurately. Moreover, our visual sensor system is more flexible in estimating the pose of different types of aircraft as it does not need to preload detailed 3D models or feature database.

In our pose estimation method, the aircraft’s structure in a single image is represented by line features, and the spatial and geometric relationships between extracted line features are explored for the extraction of aircraft structure. The line features are detected by a line segment detector (LSD) algorithm, and a mean-shift algorithm is used for locating the aircraft’s center. Based on the orientation consistency constraint of line features distributed on the fuselage, a density-based clustering algorithm is used to determine the fuselage reference line by grouping line segments with similar direction. Through the analysis of aircraft’s common wing patterns, bilateral symmetry property of aircraft, coplanar wings, and fuselage reference line are assumed. Under these prior assumptions, the line segments which correspond to the wing leading edges are extracted by vector analysis. After identifying the projected fuselage reference line and wing edges in a pair of images, line correspondences between the aircraft’s 3D structure and its 2D projection are established and the pose information is obtained using planes intersection method. As no detail 3D models and/or feature datasets are required, our vision system provides a common architecture for aircraft pose estimation and can estimate the pose of different aircraft more flexibly and efficiently.

The remainder of the article is organized as follows: Section 2 provides the coordinate system definition and an overview of our vision system. Section 3 describes our pose estimation algorithm in detail. The experimental results are presented in Section 4 to verify the effectiveness and accuracy of our algorithm. Section 5 elaborates on the conclusions.

## 2. Coordinate System Description and Problem Statement

**Figure 1:**
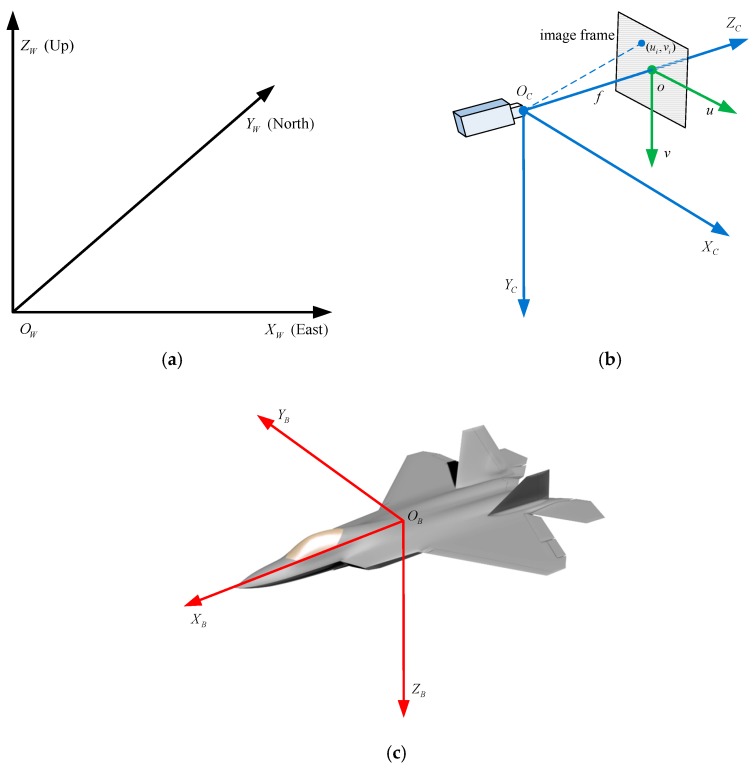
Coordinate systems: (**a**) world coordinate frame; (**b**) camera coordinate frame and image frame; (**c**) body coordinate frame.

In this section, we briefly introduce the coordinate systems and pose estimation problem. Four major coordinate systems used for pose estimation are shown in Figure 1.

Figure 1a shows the world coordinate system in which the camera tracks the aircraft, and the absolute pose of an aircraft is defined with respect to the world frame. In our method, the east–north–up (ENU) coordinate system is used as the world coordinate system.

Figure 1b shows the camera coordinate system and image coordinate system. The camera frame is tied to a monocular camera which project the aircraft onto the image frame. The optical center of the camera is treated as the origin of the camera frame, the optical axis of the camera is along the *z* axis of the camera; the horizontal axis (*u*) of the image frame is parallel to the *x* axis of the camera coordinate frame in the right direction, and the vertical axis (*v*) of the image frame is parallel to the *y* axis of the camera coordinate frame in the right-handed coordinate system.

The camera is calibrated with respect to the world coordinate system and its orientation parameters corresponding to the captured image are recorded, i.e., the interior and exterior orientation parameters of the camera are considered to be known.

Figure 1c shows the body coordinate system of an aircraft. The origin is located at the centroid of the aircraft. The *x* axis points along the fuselage reference line; the *z* axis is perpendicular to the plane containing the fuselage reference line and wing leading edge lines, direct to down side; and the *y* axis is perpendicular to the bilateral symmetry plane of the aircraft in the right-handed coordinate system. As is shown in Figure 1c, for fixed-wing aircraft, the wings are mounted to the fuselage, and the wing leading edges and fuselage reference line are approximately coplanar.

**Figure 2:**
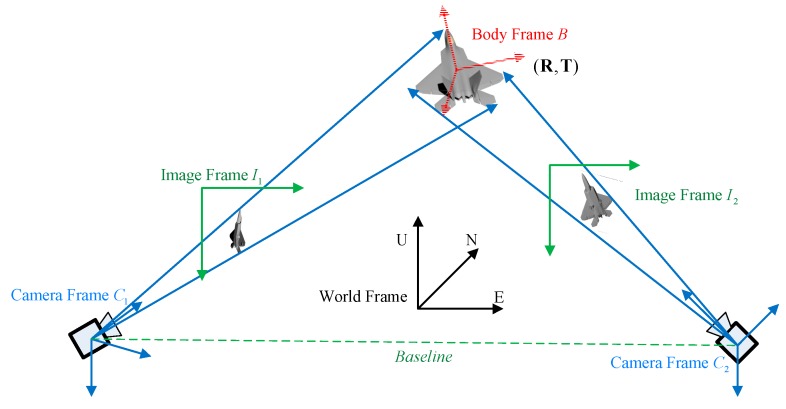
The schematic representation of the pose estimation problem.

The schematic representation of the pose estimation problem is shown in Figure 2, where a vision system using two monocular cameras is established for pose estimation of an aircraft. The baseline distance between the two cameras in our vision system is large, and our method only utilizes a wide-baseline image pair captured at the same time to solve the absolute pose of the aircraft robustly and accurately.

The location and orientation of the body frame with respect to the world frame represent the 3D pose parameters of the aircraft, which are the solutions for the pose estimation problem. For many aircraft, the bilateral symmetry is their inherent property and their wings are coplanar with the fuselage reference line. Moreover, line features distributed along the fuselage are approximately parallel to the fuselage reference line. These geometry structure features are exploited in our method to facilitate aircraft structure recognition and pose estimation.

## 3. Pose Estimation Method

For the absolute pose estimation problem, the orientation and location of the aircraft was specified using a rotation matrix R and a translation vector T which set a transformation from the body frame to the world frame. The yaw, pitch, and roll angle (the Euler angles) of the aircraft were extracted from the rotation matrix and the translation vector represented the 3D position of the aircraft in the world coordinate system.

To obtain the solution for the rotation matrix and translation vector, it was necessary to establish the correspondences between features in the body frame and the world frame. In our method, the feature correspondences were found by structure extraction. With some prior assumptions about the geometric relationships between the fuselage reference line and wing leading edges, our pose estimation method first extracted the fuselage reference line and the wing leading edge lines from a given pair of images to establish the line correspondences, then used a planes intersection method to obtain the pose information. The details of the structure extraction and plane intersection algorithm are described in the following sections.

### 3.1. Structure Extraction

A novel structure extraction method is presented to detect the fuselage reference line and wing leading edge lines from a 2D image without using 3D models, cooperative markers, or other feature datasets. For the pose estimation of a free flight aircraft at long distance, it was difficult for feature points to establish reliable correspondences due to the optical blur, ambiguities, and self-occlusions. In these cases, line features were more robust and accurate and less affected by the unpredictable motion of the aircraft. To acquire accurate and robust pose information from 2D images, line segments were used for structure description and recognition.

In our algorithm, some assumptions about the generic geometry structure features of the aircraft are explored. The most important assumptions used for structure extraction are as follows:

1. Line features distributed along the fuselage were approximately parallel to the fuselage reference line;
2. Wing leading edge lines were bilaterally symmetrical and coplanar with fuselage reference line.

These two assumptions reflect the geometry relationships between line features on the aircraft. Weak perspective projection (scaled orthographic projection), which is a first order approximation of the perspective projection, was also assumed. As the size of the aircraft is small compared to its average distance from the monocular camera, weak perspective assumption holds approximately.

Based on these assumptions, the geometric configuration of line features, such as geometric consistency (position, length and orientation) and linear combinations of vectors (directed line segments) were exploited to extract the main structure (fuselage and wing leading edges) of the aircraft and establish line correspondences. Robust and accurate structure extraction can lay a good foundation for pose estimation.

#### 3.1.1. Line Feature Detection

The line features were detected in 2D images using line segment detector (LSD) algorithm [59], which is a state-of-the-art method for extracting line segments with subpixel accuracy in linear-time. Reference [60] compared different line detection methods and made the conclusion that LSD algorithm is optimal at different illumination, scales, and blur degrees.

**Figure 3:**
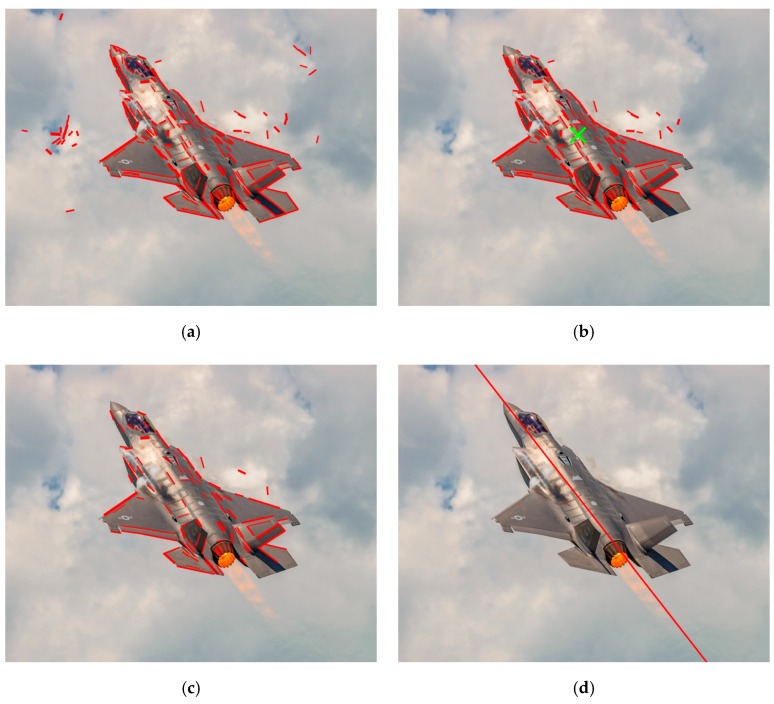
The results of line feature detection and fuselage extraction based on spatial, length and orientation consistency constraints: (**a**) line feature detection; (**b**) line clustering based on spatial consistency constraint; (**c**) length consistency constraint; (**d**) extraction of fuselage reference line based on parallel line clustering.

Figure 3a shows the result of line feature detection, in which detected line features are expressed by red line segments. As is shown in Figure 3a, the structure information of aircraft was described by line segments and the geometric configuration between these line segments was analyzed in the following sections to determine the fuselage reference line and wing leading edge lines. The set of line features detected by LSD algorithm is denoted by $S_{L}$, and the following structure extraction process is based on the line feature set $S_{L}$.

#### 3.1.2. Extraction of Fuselage Reference Line

Our structure extraction method leveraged spatial, length and orientation consistency constraints of the line features to extract the fuselage reference line. Firstly, the spatial consistency constraint was used to identify the centroid of the aircraft and combined with length constraint to remove irrelevant line features; then, orientation consistency-based line clustering analysis was performed to recognize the aircraft’s structure and estimate the direction of fuselage reference line.

In actual applications, such as flight test, take-off and landing, our vision system tracked one single aircraft and captured its 2D projected image, so the image area that contained the aircraft was a saliency region in which detected line features are concentrated. Compared to unrelated line segments distributed in the background, line segments of the aircraft were close to each other which formed a high-density region of line features. Based on this spatial consistency constraint, mean shift [61] algorithm was used for line feature clustering to determine the center of the aircraft and exclude irrelevant line features.

Mean shift is a non-parametric mode-seeking technique which locates the maxima of a density function iteratively. In our case, it was suitable for indicating the density model of line features and determining the centroid of the aircraft. Given an initial estimation, mean shift algorithm used a kernel function to determine the weight of adjacent elements and re-estimate the weighted mean of the density. The line feature set $S_{L}$ was the input of mean shift algorithm, the adjacent element were an image pixel x (represented by (u,v), where u and v are the horizontal and vertical coordinates of the pixel respectively) on a line segment in $S_{L}$, and the kernel function is a Gaussian kernel G. The Gaussian kernel G and the weighted mean m(x) are denoted as following:

(1) $$\begin{array}{l} G(x_{i}-x)=e^{-c{\left\|x_{i}-x\right\|}^{2}}\\ m(x)=(\sum_{x_{i}\in n(x)}G(x_{i}-x)\cdot x_{i})\cdot {\left(\sum_{x_{i}\in n(x)}G(x_{i}-x)\right)}^{-1} \end{array}$$

in which c represents the weight of the Gaussian kernel on the distance and n(x) is the neighborhood of adjacent elements. After the aircraft’s center is determined, line segments within a certain distance from the cluster center are retained which is considered as the line features on the aircraft, while other line segments away from the center are excluded from the set $S_{L}$. The mean shift algorithm can estimate the aircraft center robustly and has a certain tolerance to background clutter. The result of mean shift clustering is shown in Figure 3b, where the cluster center is marked by a green cross and the remaining line features are represented by red line segments. Compared to Figure 3a, we can see that a lot of irrelevant line segments distributed in the background are effectively eliminated by leveraging the spatial geometric constraint.

However, there were still some unrelated line features left in the line feature set $S_{L}$, as we can see in Figure 3b. To further reduce the adverse effect of irrelevant line segments, the length consistency constraint was adopted and only line segments longer than a certain threshold were retained in $S_{L}$.

In general, the lengths of the aircraft’s main structures (fuselage and wings) were larger than the lengths of other parts such as tail or other external mounts, so it is reasonable to exclude shorter line segments from line feature set. Moreover, the length consistency constraint improved the accuracy of structure extraction. For a line feature detected by the LSD algorithm, the uncertainty of its direction increased with the decreasing length, it means that a small pixel coordinate error of the endpoint causes greater orientation error for shorter line segments. Figure 3c shows the result of excluding shorter line segments. As can be seen, the irrelevant line features were further eliminated in comparison with Figure 3b.

After acquiring the estimation of the aircraft’s center and removing irrelevant line features, the orientation consistency constraint was utilized to estimate the direction of the fuselage reference line. Under weak perspective projection, if the line segments are parallel to each other in 3D space, then this geometric constraint of corresponding line features is invariant after the 3D world to 2D image transformation.

For many aircraft, line segments distributed along the fuselage are approximately parallel to the fuselage reference line, and the parallel world lines are transformed to the parallel image lines by the weak perspective camera. In the projected 2D image, angle values of line features along the fuselage were close to each other and concentrated around the orientation of the fuselage reference line (small standard deviation). Moreover, compare with other parts of the aircraft, fuselage contains most parallel line segments. By performing parallel line clustering to represent the aircraft structure, the orientation of the fuselage reference line was determined.

The input of the parallel line clustering analysis was the directions of line features in $S_{L}$, which is represented as $\Theta =\left\{\theta_{1},\theta_{2},\ldots ,\theta_{i},\ldots ,\theta_{N}\right\}$. Based on the orientation consistency constraint, the center of the highest density area of Θ indicates the orientation of the fuselage. The orientation of a line feature is defined as the angle $\theta_{i}$ of the straight line specified by this line feature, and the orientation of the fuselage reference line is represented as $\theta_{f}$. To seek regions in Θ which have a high density and estimate the direction of the fuselage reference line, density-based spatial clustering of application with noise (DBSCAN) [62] algorithm was used for the parallel line clustering.

**Figure 4:**
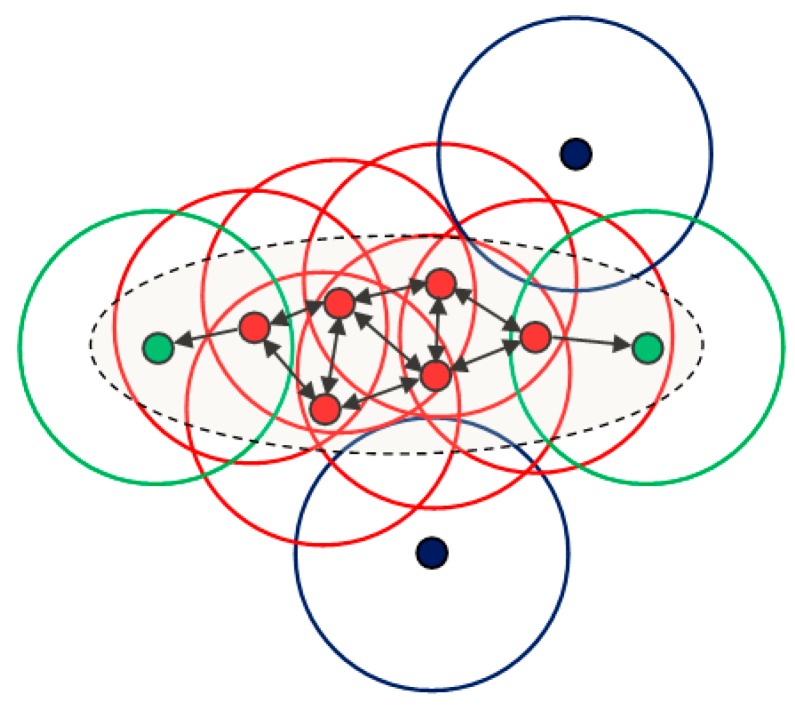
The schematic representation of the density-based spatial clustering of application with noise (DBSCAN) algorithm: the red points indicate the core points, the green points indicate the border points, the blue points indicate the outliers, and the gray ellipse indicates the cluster (core points and border points). The radius of the circle represents the distance threshold.

DBCSAN is a density-based clustering algorithm, and the clustering solely depends on the spatial density mode of the data. Two parameters were used in the DBSCAN algorithm to describe the spatial distribution of the data: the distance threshold ε and the minimum number of points minPts. The distance threshold ε measured the proximity of two data points while minPts determines the minimum number of line features required to form a high-density region. For parallel line clustering, ε is the absolute difference between angle values. By using these two parameters, data points are partitioned into three types according to their spatial patterns, as is shown in Figure 4:

- Core points (red points in Figure 4, the dense region of a cluster): its ε neighborhood contains at least minPts points;
- Border points (green points in Figure 4, the edge region of a cluster): the number of data points in its ε neighborhood is less than minPts, but it can be reached from a core point in its ε neighborhood (as displayed by one-way arrows in Figure 4);
- Noise points (blue points in Figure 4, isolated outliers): it is neither a core point nor a border point.

To discover the orientation consistent clusters in Θ, the DBSCAN algorithm first counted the number of points in every data point’s ε neighborhood and identifies the core points according to minPts; then core points were partitioned into parallel line clusters leveraging the geometric constraint on the connected graph (see two-way arrows in Figure 4). Finally, for every non-core point, if there was a core point in its ε neighborhood, it was considered as a border point, otherwise, it was a outlier. The orientation-consistent clusters with most parallel lines was considered as the representation of the fuselage in the image, and the orientation information of the fuselage reference line was acquired by extracting the center of this line feature cluster. Compared to other clustering approaches such as *k*-means [63] and Gaussian mixture model (GMM) [64], the DBSCAN algorithm did not need prior knowledge about the number of clusters and can discover high density areas of arbitrary shape and size. It was also insensitive to outliers and the ordering of the data points.

After obtaining the orientation-consistent cluster which represents the fuselage structure, line features not belonging to the fuselage are excluded from $S_{L}$ and form a new set $S_{W}$ which was used for the extraction of wing leading edges. As only line features belonging to the fuselage remained in $S_{L}$, it was possible to identify the fuselage’s center with a higher precision, and the image moment method was used to re-estimate the center of $S_{L}$ to obtain the estimation of the fuselage center, which is given by:

(2) $${\tilde{u}}_{m}=\frac{\sum_{i=1}^{N}u_{i}}{N}\;\;{\tilde{v}}_{m}=\frac{\sum_{i=1}^{N}v_{i}}{N},$$

where $\left({\tilde{u}}_{m},{\tilde{v}}_{m}\right)$ is the estimated center of the fuselage, and N is the number of pixels on the line features in $S_{L}$. In the case of a clean background, the image moment method can also be used to estimate the centroid of the aircraft. The result of parallel line clustering is shown in Figure 3d, in which the estimated centroid of the fuselage is marked by a green cross and the fuselage reference line is indicated by the red line. As is shown in Figure 3d, the fuselage reference line was correctly extracted. The estimated orientation $\theta_{f}$ and center $\left({\tilde{u}}_{m},{\tilde{v}}_{m}\right)$ determine the 2D pose information of the aircraft’s fuselage in the image, and the extracted fuselage line will provide important auxiliary information for the following structure extraction.

#### 3.1.3. Extraction of Wing Leading Edges

To identify the line features which correspond to the wing leading edge lines of the aircraft, it was assumed that the wings were bilaterally symmetrical and approximately coplanar with the fuselage reference line (the fuselage reference line passes through the point of intersection of the wing leading edge lines). By observing the wing patterns of many aircraft, the validity of the assumption was confirmed. Based on the lateral symmetry of the aircraft, the wing leading edges were expressed as two symmetric vectors of equal length. With the additional condition that the wings were coplanar with the fuselage reference line, the sum of these two vectors was parallel to the fuselage reference line.

Since the geometric properties of linear combinations of vectors are invariant under weak perspective projection, after the aircraft in three-space is projected onto the image plane by a weak perspective camera, the addition of two directed wing leading edges in the image is still parallel to the fuselage reference line. The following is a proof of this geometric invariant property.

In the world coordinate frame, two vectors representing the wing leading edges were denoted by $\vec{OA}$ and $\vec{OB}$; the sum of these two vector which is parallel to the fuselage reference line is $\vec{OC}$. While in the image coordinate frame, the corresponding projection vectors were represented as $\vec{oa}$, $\vec{ob}$ and $\vec{oc}$. The world coordinates of a vector endpoint were denoted by $X_{i}={\left(X,Y,Z,1\right)}^{T}$ and the corresponding image coordinates were $x_{i}={\left(u,v,1\right)}^{T}$. As the world and image points ($X_{i}$ and $x_{i}$) are represented as homogeneous vectors, and depth of field is a positive constant under weak perspective projection, the world to image transformation of every vector endpoint can be represented compactly as:

(3) $$\{\begin{array}{c} x_{o}=PX_{O}\\ x_{a}=PX_{A}\\ x_{b}=PX_{B}\\ x_{c}=PX_{C} \end{array},$$

where P is the projection matrix of the camera. By using Equation (3), the geometric transformations between vectors in the world frame and image frame are established as follows:

(4) $$\{\begin{array}{c} \vec{oa}=P\left(X_{A}-X_{O}\right)=P\vec{OA}\\ \vec{ob}=P\left(X_{B}-X_{O}\right)=P\vec{OB}\\ \vec{oc}=P\left(X_{C}-X_{O}\right)=P\vec{OC} \end{array}.$$

As $\vec{OC}$ is the addition of $\vec{OA}$ and $\vec{OB}$:

(5) $$\vec{OA}+\vec{OB}=\vec{OC}.$$

By substituting Equation (5) into Equation (4), the geometric invariant property is obtained:

(6) $$\vec{oa}+\vec{ob}=\vec{oc}.$$

**Figure 5:**
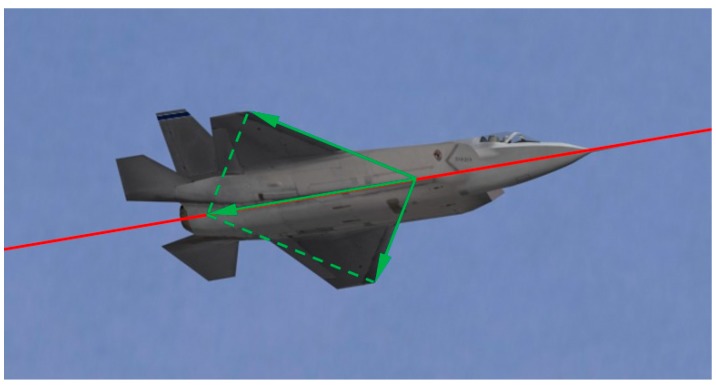
The geometric constraint of the directed wing leading edges and fuselage reference line in the image frame.

Since $\vec{oc}$ is the projection of $\vec{OC}$, the sum of $\vec{oa}$ and $\vec{ob}$ is parallel to the fuselage reference line in the image. An intuitive representation of this geometric constraint in the image frame is shown in Figure 5, where the red line indicates the fuselage reference line and the green arrows indicate the directed wing leading edges. After the 3D world to 2D image transformation under weak perspective projection, the addition of directed wing leading edges is still parallel to the unit vector of the fuselage reference line.

Based on this geometric invariant property, the wing leading edge lines in the image are extracted using vector calculations. We first vectorized line segments in $S_{W}$ based on the extracted fuselage reference line. For the vector representation of every line segment, the endpoint of the line segment which was father from the fuselage reference line was decided as the destination of the vector, and by extending original line segment to an intersection with the fuselage reference line, the intersection is determined as the beginning of the vector. Then for every two vectors in the set $S_{W}$, vector calculations were performed to obtain the value of an objective function. The objective function was proposed to extract the wing leading edge lines based on the geometric invariance constraints. Let the unit vector giving the direction of the fuselage reference line be $v_{f}$, for two vectors $v_{a}$ and $v_{b}$ in $S_{W}$, whose beginnings are $x_{a}$ and $x_{b}$ respectively, the objective function is given by:

(7) $$\begin{array}{l} F(v_{a},v_{b})=w_{1}f_{\theta_{1}}+w_{2}f_{\theta_{2}}+w_{3}f_{M_{1}}+w_{4}f_{M_{2}}+w_{5}f_{M_{3}}\\ f_{\theta_{1}}=\frac{(v_{a}+v_{b})\cdot v_{f}}{\left|v_{a}+v_{b}\right|}\\ f_{\theta_{2}}=\frac{1}{2}\cdot \left|\frac{v_{a}}{\left|v_{a}\right|}+\frac{v_{b}}{\left|v_{b}\right|}\right|\\ f_{M_{1}}=\left|v_{a}-v_{b}\right|\\ f_{M_{2}}=-\left|x_{a}-x_{b}\right|\\ f_{M_{3}}=\left|v_{a}\right|+\left|v_{b}\right|\\ w_{i}\geq 0,\;\sum_{i=1}^{5}w_{i}=1 \end{array}$$

where |·| represents the magnitude of a vector and each item in the objective function is normalized into the range [0, 1]. The two vectors for which $F(v_{a},v_{b})$ attains its maximum value are regarded as the correspondences of the wing leading edges and used to establish the line correspondences, i.e., our method extracts the wing leading edges from the image by solving this:

(8) $$\underset{v_{a},v_{b}\in S_{W}}{\arg\max}F(v_{a},v_{b}):=\{v_{a},v_{b}|v_{a},v_{b}\in S_{W}\wedge \forall v_{i},v_{j}\in S_{W}:F(v_{i},v_{j})\leq F(v_{a},v_{b})\}$$

The parameter $f_{\theta_{1}}$ is the cosine of the angle between $v_{a}+v_{b}$ and $v_{f}$, and measures the degree of parallelism between two vectors. If $v_{a}+v_{b}$ is parallel to the fuselage reference line, then $f_{\theta_{1}}$ reaches its maximum, i.e., $f_{\theta_{1}}=1$. For two vectors corresponding to the wing leading edges, the addition is approximately parallel to the fuselage reference line, and the value of $f_{\theta_{1}}$ is close to 1.

The parameter $f_{\theta_{2}}$ represents the cosine of half-angle between $v_{a}$ and $v_{b}$. For the wing trailing edges of an aircraft, the addition of corresponding vectors is also parallel to the fuselage reference line, so $f_{\theta_{2}}$ is used to distinguish between the wing leading edges and trailing edges. As the angle between the wing leading edges is usually smaller than the angle between the wing trailing edges, $f_{\theta_{2}}$ gains a greater value for the wing leading edges.

The parameter $f_{M_{1}}$ is the magnitude of $v_{a}-v_{b}$. As the wingspan dimension represents the width of an aircraft, for $v_{a}$ and $v_{b}$ corresponding to the wing leading edges, $f_{M_{1}}$ indicates the distance between wingtips and reaches its largest value.

The parameter $f_{M_{2}}$ measures the distance between $x_{a}$ (the beginning of $v_{a}$) and $x_{b}$ (the beginning of $v_{b}$). With the wing leading edges intersecting at a point, if $v_{a}$ and $v_{b}$ corresponds to the wing leading edge lines, then the value of $f_{M_{2}}$ is close to zero.

The parameter $f_{M_{3}}$ is the sum of $\left|v_{a}\right|$ and $\left|v_{b}\right|$. Since the wing leading edge in general have a larger length than aircraft’s other structures, including the wing trailing edge, $f_{M_{3}}$ has a larger value for the vectors corresponding to the wing leading edges.

Based on the above analysis, $F(v_{a},v_{b})$, the weighted sum of these items, attained its maximum at the two vectors corresponding to the wing leading edges empirically. Among these items, $f_{\theta_{1}}$, $f_{M_{1}}$ and $f_{M_{2}}$ play more important roles and the corresponding weights are greater. Although the 2D pose information of the fuselage was required, the extraction of the wing leading edges can tolerate the error of fuselage extraction to some extent and a very high-precision fuselage pose estimation is not necessary.

**Figure 6:**
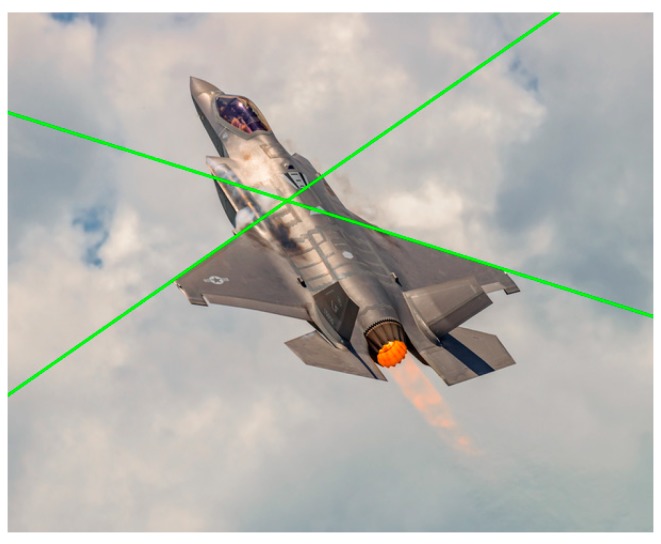
The result of wing leading edge extraction.

By solving Equation (8), the lines which the wing leading edges belong to were detected in the image and the correspondences between line features in the image and aircraft structure in 3D space are established. The results of wing leading edge extraction are shown in Figure 6 where the green lines indicate the wing leading edge lines of the aircraft. As is shown, the wing leading edge lines were extracted correctly.

While the wing trailing edge could also be extracted by adjusting the weights in Equation (7), line features of the wing trailing edges were vulnerable to the interference from some other parts of the aircraft. In Figure 6, the wing trailing edge was occluded by the tail and it was difficult to detect corresponding line features. Considering line features of the wing leading edges were less affected by the unpredictable motion of the aircraft and more stable, only the wing leading edge lines are extracted for pose estimation.

### 3.2. Planes Intersection Based on Line Correspondences

**Figure 7:**
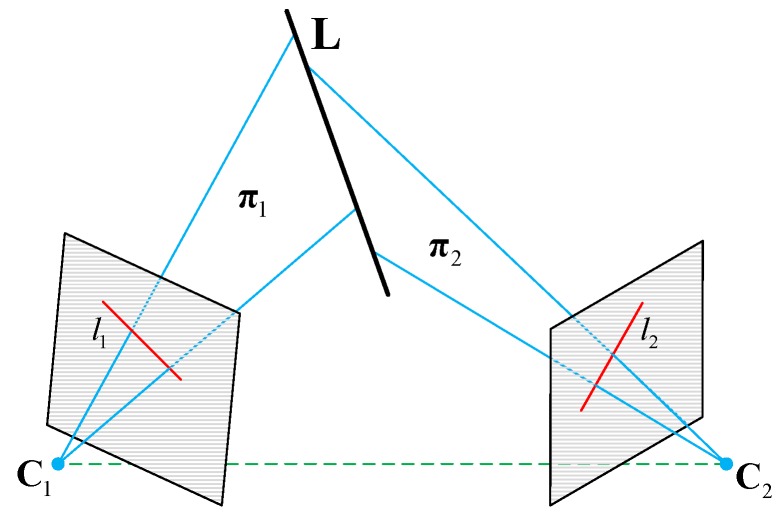
Geometric configuration of plans intersection.

After extracting the wing leading edge lines and establishing the line correspondences, the planes intersection method was used for aircraft pose estimation. The geometric configuration of the planes intersection method is explained in Figure 7. As we can see, the representation of a line in three-space (the wing leading edge line in our method) was determined by the two planes defined by line correspondences.

Although the fuselage reference line was extracted robustly and used to facilitate the wing leading edge extraction, the parameters which identify the fuselage reference line may be less accurate. To perform high-precision pose estimation, only wing leading edge lines in an image pair were utilized in intersection calculation.

In Figure 7, two monocular cameras are used for plane-plane intersection and indicated by their optical centers $C_{1}$ and $C_{2}$, and by image planes (gray regions in Figure 7). Similar to Equation (3), the camera model under weak perspective projection is represented compactly as:

(9) $$x=P_{i}X,$$

in which $P_{i}\;(i=1,2)$ is the projection matrix of the monocular camera $C_{i}$, $X={\left(X,Y,Z,1\right)}^{T}$ is the world coordinates and $x={\left(u,v,1\right)}^{T}$ is the corresponding image coordinates.

Our planes intersection method considered the lines as infinite and the endpoints of line features were not used for pose estimation. Let the 3D line L in the world frame represent one of the wing leading edge line, and $l_{i}$ be the parametric representation of L’s projected line on the image frame. The parameters of L in three-space can be computed by intersecting the planes $\pi_{1}$ and $\pi_{2}$.

The plane $\pi_{i}$ is defined by the image line $l_{i}$ and the optical center $C_{i}$, and it can be represented conveniently as a 4-vector:

(10) $$\pi_{i}^{T}=l_{i}^{T}P_{i},$$

where $l_{i}$ and $P_{i}$ are already known, and for a world point X contained in the plane $\pi_{i}$, $\pi_{i}^{T}X=0$. Based on Equation (10), the 3D line L, which is the intersection of the two planes $\pi_{1}$ and $\pi_{2}$, is parametrized by the span representation:

(11) $$L=\left[\begin{array}{c} \pi_{1}^{T}\\ \pi_{2}^{T} \end{array}\right]=\left[\begin{array}{c} l_{1}^{T}P_{1}\\ l_{2}^{T}P_{2} \end{array}\right].$$

Here, the line L is represented as a 2×4 matrix, and if a world point lies on the line, then LX=0. After obtaining the span representation of L in 3D space, the normalized vector v giving the direction of L is also determined. Our pose estimation method uses the normalized vector v to identify the orientation of a wing’s leading edge in the world frame, and obtain the 3D attitude of the aircraft.

Let $L_{wr}$ be the right wing leading edge line in 3-space, and $L_{wl}$ be the left wing leading edge line; The normalized vectors of $L_{wr}$ and $L_{wl}$ are represented as $v_{wr}$ and $v_{wl}$ respectively. The direction of the *x* axis of the body frame is parallel to $-(v_{wr}+v_{wl})$, while the direction of the *y* axis of the body frame is parallel to $v_{wr}-v_{wl}$. By using singular value decomposition (SVD) [65], the rotation matrix between the body frame and world frame which determines the aircraft attitude in three-space is obtained up to a reflective ambiguity.

To avoid this reflective ambiguity, an initial pose constraint is used in our method. The approximate orientation of the aircraft is specified in the initial frame of the image sequence, and the orientation information of the current frame was used in the next frame. In practice, the approximate value of the roll angle of the aircraft was provided, or the approximate position of one wing tip was marked on one image of the initial image pair. It was easy to provide this pose constraint in the application scenarios of our pose estimation method, for example, take-off, landing, and flight testing.

The point of intersection of $L_{wr}$ and $L_{wl}$ indicates the translation vector of the body frame with respect to the world frame. Based on the span representation of $L_{wr}$ and $L_{wl}$, overdetermined equations are established to compute the world coordinates of the point of intersection as follows:

(12) $$\begin{array}{l} AX=0\\ A=\left[\begin{array}{c} L_{wr}\\ L_{wl} \end{array}\right] \end{array}$$

in which X is the point of intersection (a homogeneous 4-vector) of $L_{wr}$ and $L_{wl}$, and A is a 4 × 4 matrix. By performing a factorization of matrix A via the SVD, the singular vector corresponding to the smallest singular value of A is the solution of the overdetermined equations AX=0. In addition, based on the epipolar constraint, an optimal estimator for the point of intersection on the image plane can be used to reduce the geometric errors before the matrix factorization [66].

As the normalized vectors $v_{wr}$ and $v_{wl}$ determine the rotation matrix and the point of intersection of $L_{wr}$ and $L_{wl}$ identifies the translation vector, the transformation from the body frame to the world frame which solves the aircraft pose is acquired. Based on the line correspondences, the planes intersection method obtained the pose information of model-unknown aircraft. Moreover, our pose estimation method can be easily extended to multi-camera systems.

### 3.3. Algorithm Pipeline

The pipeline of our pose estimate method is summarized in this section, as is shown in Algorithm 1.
**Algorithm 1.** Pose estimation based on geometry structure features and line correspondences.Input:A pair of images captured at the same time, the parameter matrices of the two cameras $P_{1}$, $P_{2}$, and the initial pose constraint.Output:The pose information of the aircraft.Step 1Detect line features in images using the LSD algorithm;Step 2Estimate the location of the aircraft’s center in images and eliminate irrelevant line features based on the spatially and length consistency constraints;Step 3Determine the orientation of fuselage reference line using parallel line clustering;Step 4Extract the wing leading edge line in the image pair based on generic geometry structure features of aircraft and vector analysis;Step 5Acquire the pose information of the aircraft by using the planes intersection method.

## 4. Experiments and Results

The effectiveness and accuracy of our method have been evaluated by experiments on real and synthetic images. The performance of our structure extraction method was verified by real images of various types of aircraft, and synthetic images of different aircraft at different viewpoints and real images captured in ground laboratory experiments were used to measure the accuracy and robustness of the pose estimation algorithm. Our pose estimation method was implemented by MATLAB based on a laptop with an Intel Core i7 CPU with a 2.80 GHz processor and 8.00 GB of RAM.

### 4.1. Structure Extraction Results

This section provides qualitative evaluation of our structure extraction method by using real images downloaded from the Internet. There were 50 total real images which contain a variety of aircraft, and some of these images were difficult as they contained self-occlusion, cluttered backgrounds, disruption of external mounts, or perspective effects.

**Figure 8:**
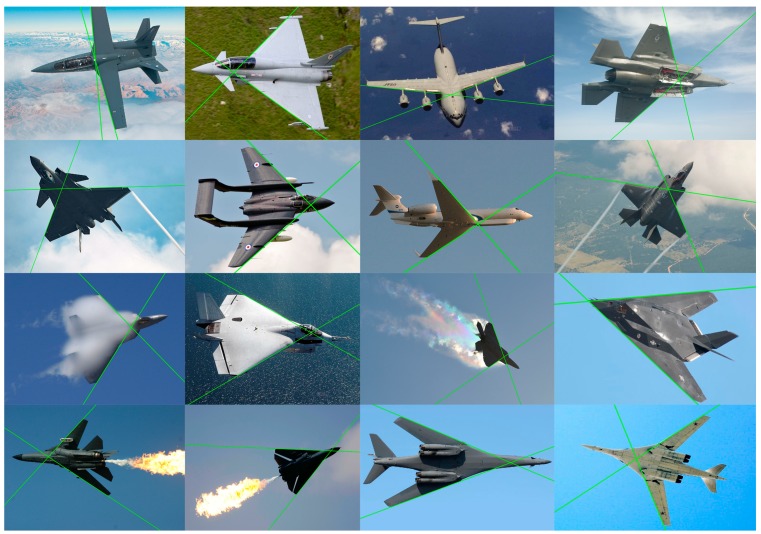
The results of the wing leading edge extraction.

Since only wing leading edges were used to solve the pose estimation problem, the performance of the structure extraction method was evaluated by the correctness of the extracted wing leading edge lines. Our approach can cope with a wide variety of aircraft flexibly and does not rely on detailed 3D models or cooperative marks. In the experiment, the wing leading edge lines in 42 of the 50 images were correctly extracted. Some of the results are shown in Figure 8.

As is shown in Figure 8, our method has the ability of suppressing the interference of outliers and identifies the wing leading edge line robustly and effectively. In addition, our method can acquire the structure information of a variable-sweep aircraft (see bottom row of Figure 8). For an aircraft with flexible wings (e.g., F111, B-1B), the estimation of its pose was very hard for model-based methods, while our approach can handle it with no need of modifying the algorithm. For the aircraft that were difficult to accurately determine the fuselage structure, our method can tolerate the estimation errors of the fuselage orientation and aircraft center and detect the wing leading edges correctly.

**Figure 9:**
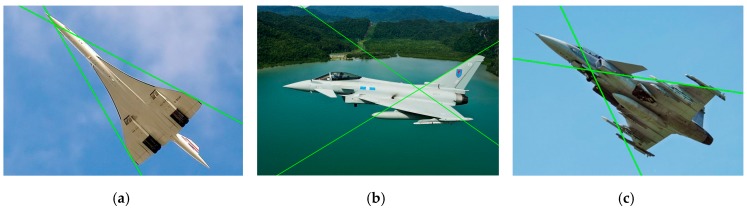
Incorrect results of the extraction of wing leading edge lines: (**a**) the leading edge of the wing is not a straight line; (**b**) portions of the wings are not visible in aircraft images from some viewpoints; (**c**) some parts of the aircraft affect the line detection.

Some incorrect wing extraction results of our approach are shown in Figure 9. There are some reasons for these incorrect results:

1. The leading edge of the wing is not a straight line (see Figure 9a), i.e., the aircraft structure (wing patterns or fuselage structure) did not meet our assumptions about the generic geometry features.
2. Portions of the wings are not visible in aircraft images from some viewpoints (see Figure 9b). Under some poses, the wings are occluded by fuselage or other parts of the aircraft which makes our method unable to extract the wing leading edge lines. This situation is not usual for the long-range measurements of our vision system located on the ground.
3. Some parts of the aircraft (external mounts) or other interference factors (changing weather or light conditions) affect the line detection, i.e., some unreliable line features are extracted and affect structure extraction (see Figure 9c).

Although our algorithm was developed for long-range measurements in which the assumption of weak perspective projection holds, the experimental results show that our algorithm can tolerate a certain perspective effect. The assumptions about the generic geometry structure feature of aircraft were also not expected to hold strictly. Even if the wing leading edge lines were not strictly coplanar with the fuselage reference line, our method is still capable of correctly extracting the wing leading edges.

### 4.2. Pose Estimation Results

Synthetic images of different aircraft and real images captured in ground laboratory experiments have been used to test the performance of the method. In experiments, our pose estimation algorithm is compared with Li’s method [32] which is a binocular vision method based on stereo matching. In Li’s method, the feature points obtained by the line feature detection were used for stereo matching, and the pose information of a non-cooperative target was acquired based on the triangulation method and 3D reconstruction. Since the triangulation method was widely adopted for pose estimation in classical binocular vision methods, and the pose estimation pipeline of Li’s method was also commonly used, it was chosen as the comparison method to evaluate the performance of our proposed method.

The algorithms were evaluated in pose estimation errors. For any given ground truth aircraft pose ($R_{true}$ and $T_{true}$) and corresponding pose estimates ($\hat{R}$ and $\hat{T}$), the rotation error is defined by $error_{rot}=\left\|\hat{\theta }-\theta_{true}\right\|$ where $\hat{\theta }$ and $\theta_{true}$ are the rotation angles of $\hat{R}$ and $R_{true}$, respectively, and the translation error is defined by $error_{trans}=\left\|\hat{T}-T_{true}\right\|$.

#### 4.2.1. Experiments on Synthetic Images

In this section, given detailed 3D models of different aircraft, synthetic image pairs were created to evaluate our pose estimation method and compared algorithm.

**Figure 10:**
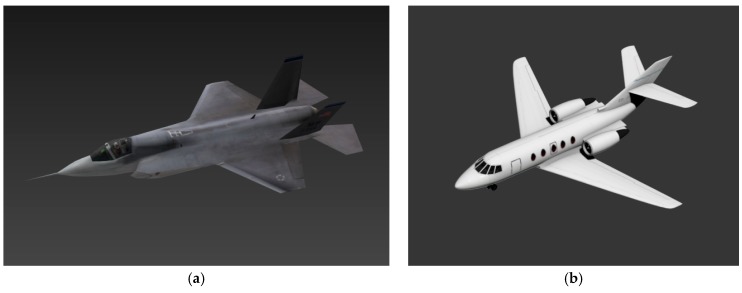
Two aircraft models: (**a**) F35 model; (**b**) Cessna model.

Figure 10 shows the two models used in simulation experiments. These two full-size models were downloaded from [67], the aircraft model shown Figure 10a is the simulation of a F35 fighter (hereinafter referred to as F35), and the model shown in Figure 10b represents a Cessna Citation commercial airplane (hereinafter referred to as Cessna). The size of the F35 model is 8.97 m × 15.16 m × 3.01 m (length, width, height), and the size of Cessna model is 21.07 m × 22.13 m × 6.88 m (length, width, height).

Autodesk 3ds Max [68], a software for 3D modeling, rendering and visualization, was used to simulate our vision system and generate the synthetic images for pose estimation based on its rendering engine. Three simulation scenarios were established to test the algorithms. In each scene, two cameras were created to track the aircraft and capture its 2D projected image pairs. The world coordinate used in experiments was the east–north–up (ENU) coordinate system, and the interior and exterior orientation parameters of the two cameras in the world coordinate system were known. Table 1 shows the detailed information about these three scenes.

As is shown in Table 1, the baseline distances between the cameras were large and cameras with different interior parameters were used to generate the wide-baseline image pairs. The baseline distances in the three scenes were 1059.48 m, 1923.54 m and 300 m respectively.

The F35 model was used in scenes 1 and 2, while the Cessna model was used in scene 3. To verify the performance of our pose estimation algorithm, different kinds of aircraft motion were simulated in these scenes. For aircraft attitude simulation, the aircraft model was rotated around the x, y, and z axes of the body coordinate frame to imitate the changes of the roll angle γ, pitch angle ψ, and yaw angle φ, respectively, and the angle range was determined according to the actual flight situations.

In scene 1, the ground truth translation vector of F35 model was $T_{true}=(0,0,500\;m)$ while different attitudes were simulated. Table 2 shows the attitude of F35 model. For the rotation angles $\left(\theta_{x},\theta_{y},\theta_{z}\right)$ in Table 2, $\theta_{x}$ indicates the roll angle, $\theta_{y}$ indicates the pitch angle, and $\theta_{z}$ indicates the yaw angle. As we can see from Table 2, 13 image pairs were generated for pose estimation.

For scenes 2 and 3, aircraft trajectory flight was modeled and simulated. In scene 2, the translation vector of F35 model was $T_{true}=(x,0,500\;m)$, where x ranged from –500 m to 500 m by a step of 100m; the rotation angles were (0,0,90°). In scene 3, the translation vector of Cessna model was $T_{true}=(0,y,150\;m)$, where y ranged from –100 m to 0 by a step of 10 m; the rotation angles were $\left(0,\theta_{y},0\right)$, where $\theta_{y}$ varied from 15° to 25° by a step of 1°. There are 11 pairs of images rendered in Scene 2 and Scene 3 respectively.

**Figure 11:**
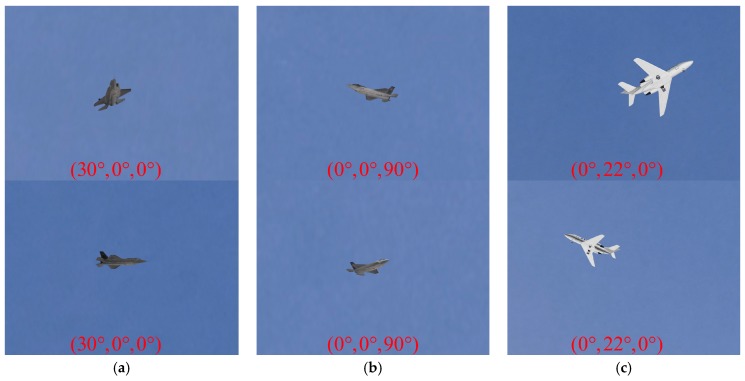
Synthetic image pairs generated in different scenes: (**a**) an image pair of F35 model in scene 1; (**b**) an image pair of F35 model in scene 2; (**c**) an image pair of Cessna model in Scene 3.

In our experiments, 35 total pairs of images were synthesized, and some of these images are shown in Figure 11. In Figure 11, every column (Figure 11a–c) represents an image pair captured at the same time in scenes 1–3. The top row indicates the synthetic images of aircraft (F35 and Cessna) in different scenes generated by camera 1, while the bottom row represents the synthetic images captured by camera 2. As we can see from Figure 11, natural light and sky backgrounds were simulated to make the flight scenarios more realistic, and the rotation angles $\left(\theta_{x},\theta_{y},\theta_{z}\right)$ of the aircraft are also shown in every image. The indexes of the image pairs in Figure 11a–c are 13, 1, and 8 in the corresponding scenes respectively.

For the wide-baseline image pairs used in our experiments, aircraft from different viewpoints had different scales, attitudes and self-occlusion which increases the difficulty in finding corresponding features. Moreover, the optical blur caused by long-distance imaging may also affect the structure extraction. These factors make it quite challenging for pose estimation methods to establish reliable and accurate feature correspondences.

**Figure 12:**
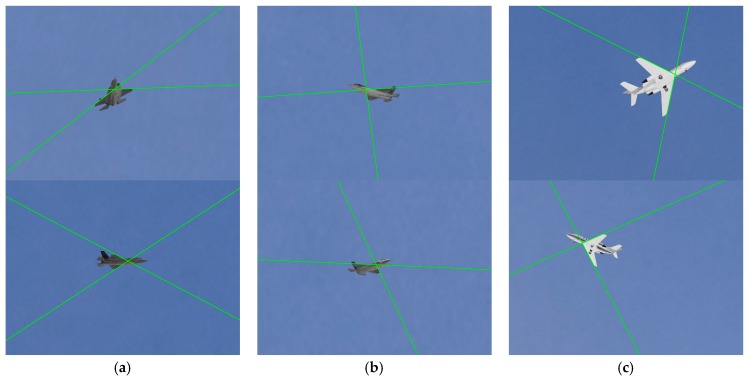
Wing leading edge line extraction results: (**a**) the results on an image pair of F35 model in scene 1 (Figure 11a); (**b**) the results on an image pair of F35 model in scene 2 (Figure 11b); (**c**) the results on an image pair of Cessna model in scene 2 (Figure 11c).

The structure extraction results on the image pairs in Figure 11 are shown in Figure 12, where the wing leading edge lines are indicated by the green lines. In our experiments, the wing leading edges in all synthetic images were extracted correctly by our structure extraction method which further verify the robustness and effectiveness of our method. Only when the aircraft wing structure is extracted robustly and accurately, can the method lead to high precision pose estimates.

As it was difficult for Li’s method to perform effective and reliable feature matching in these wide-baseline image pairs, the structure extraction results of our method were used to facilitate determining outliers and establishing effective feature point correspondences for Li’s method. The aircraft models were also used in Li’s method to solve the absolute pose for the comparison, while our algorithm estimates the aircraft pose with no need of these 3D models.

**Figure 13:**
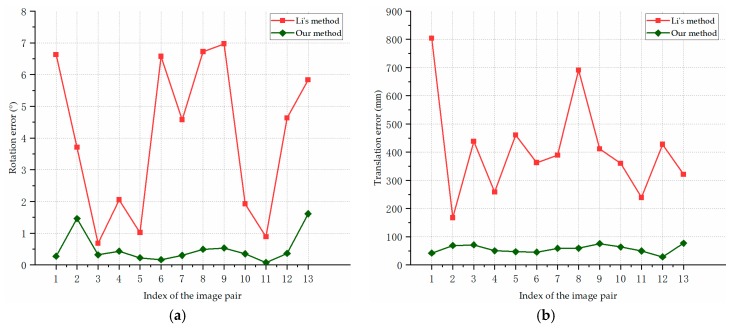
Pose estimation errors for the synthetic images of F35 model in Scene 1: (**a**) rotation errors; (**b**) translation errors.

Figure 13 shows the pose estimation errors of our algorithm and Li’s method on the synthetic images of F35 model in scene 1. Figure 13a reports the rotation errors, Figure 13b represents the translation errors. As is shown in Figure 13, our pose estimation method performed consistently better than the compared method in the estimation of the rotation angle and translation. For the aircraft with different attitudes, our method obtained stable and accurate pose estimation results. The average rotation error is 0.51°, and the average translation error is 56.85 mm.

**Figure 14:**
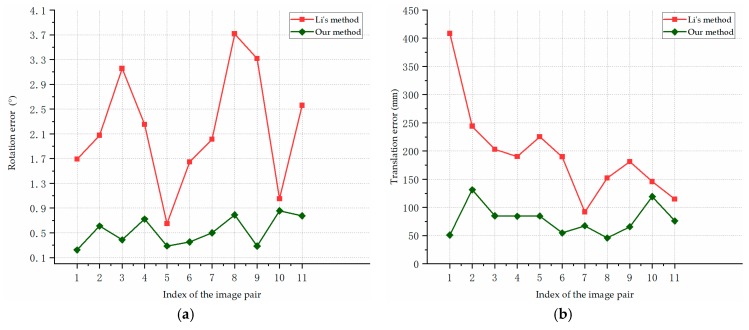
Pose estimation errors on the synthetic images of F35 model in Scene 2: (**a**) rotation errors; (**b**) translation errors.

Figure 14 shows the pose estimation errors of our algorithm and the compared method on the synthetic images of F35 model in scene 2. Figure 14a presents the rotation errors, and Figure 14b shows the translation errors. In Figure 14, our method was more accurate than the compared method in pose estimation in all the cases. While the baseline and measurement range were larger in scene 2, our algorithm still estimated the aircraft pose with a high precision. The average rotation error of our method in scene 2 was 0.53°, and the average translation error was 78.73 mm.

**Figure 15:**
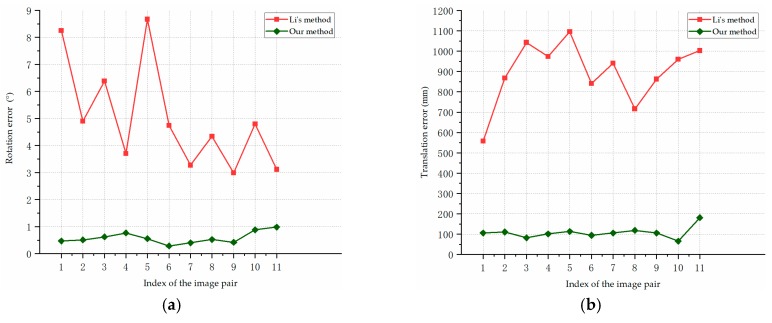
Pose estimation errors on the synthetic images of Cessna model in Scene 3: (**a**) rotation errors; (**b**) translation errors.

Figure 15 shows the pose estimation errors of our algorithm and the compared method on the synthetic images of Cessna model in Scene 3. Figure 15a reports the rotation error, and Figure 15b presents the translation errors. As we can see from Figure 15, the stability and accuracy of our proposed method was significantly higher than the compared method, and a more complex motion of the aircraft in scene 3 almost has no impact on the accuracy of our algorithm. The average rotation error of our method was 0.58°, and the average translation error was 107.72 mm.

In contrast with Li’s method, our algorithm estimates the pose of aircraft more accurately and robustly. In most cases, the rotation angle errors of our method were within 1°, and the translation errors were around or less than 0.1 m. As is shown in Figure 13, Figure 14 and Figure 15, the result curves of Li’s method fluctuated severely, which indicate that the triangulation process is sensitive to noises and different experimental conditions. The triangulation method estimated the pose information based on the point of intersection of two lines; and its measurement uncertainty increased with a larger measurement distance which makes the results more sensitive to noises. Although cameras with different parameters and baselines were used, our approach performed stably in different scenes, which indicates the stability and accuracy of our structure extraction and planes intersection methods. The experimental results suggest that our approach can achieve robust and accurate pose estimation for different types of aircraft.

#### 4.2.2. Ground Laboratory Experiment

**Figure 16:**
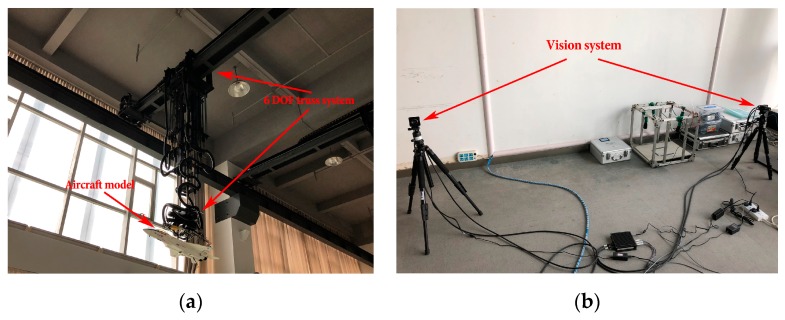
The set-up for our ground laboratory experiment: (**a**) the aircraft model and six degree of freedom (DOF) truss system; (**b**) the vision system consisting of two calibrated and synchronized monocular cameras.

In this section, the ground laboratory experiment was conducted to evaluate the accuracy of our algorithm on real images. Figure 16 shows the setup of our ground laboratory experiment.

As we can see from Figure 16a, there are an aircraft model and a six degree of freedom (DOF) truss system for experimental validation. The aircraft model in Figure 16a is a model of the real aircraft at a smaller scale (about 1/20), whose size is 987 mm × 658 mm × 285 mm (length, width, height). The aircraft pose is recorded by the six DOF truss system.

The vision system used in the experiment consisted of two calibrated and synchronized monocular cameras with a baseline of 2.8 m (see Figure 16b). The focal length of the camera lenses was 35 mm, the captured image resolution was 1280 × 960 pixels, and the world coordinate system coincides with the left camera coordinate system. In our laboratory experiment, the aircraft model moved from (0.1 m,0.4 m,12.0 m) to (0.4 m,0.1 m,8.0 m) at a constant speed; the rotation angles were (0,10°,0); and there were totally 13 pairs of images captured by the vision system.

**Figure 17:**
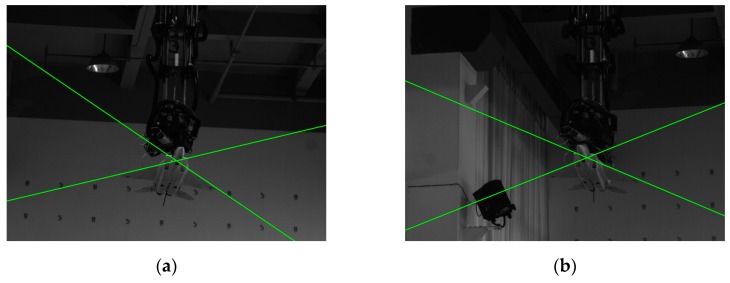
Wing leading edge extraction results on a real image pair of the aircraft model: (**a**) the real image captured by the left camera; (**b**) the real image captured by the right camera.

The results of wing leading edge extraction on the real image pairs are shown in Figure 17, where the wing leading edge lines are represented by the green lines. Our structure extraction method extracted the wing leading edges in all real image pairs correctly.

**Figure 18:**
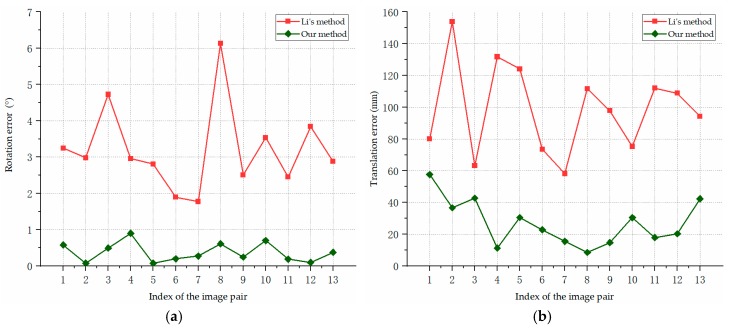
Pose estimation errors for the real images of the aircraft model in laboratory experiment: (**a**) rotation errors; (**b**) translation errors.

Figure 18 shows the pose estimation errors of our algorithm and Li’s method on the real images captured by our vision system. The rotation errors are shown in Figure 18a, and the translation errors are reported in Figure 18b.

As we can see from Figure 18, the experimental comparison shows that our method achieved more stable and accurate pose estimates. The average rotation error was 0.37°, and the average translation error was 27.01 mm. The results in Figure 18 further validate the performance of our pose estimation method on the real images.

Considering that the vision system was based on two monocular cameras, in the imaging process, the lens aberration of cameras will introduce geometric distortion, such as barrel distortion or pincushion distortion, which reduced the accuracy of line detection, and these inaccurate line features increased errors of planes-intersection and pose estimation. To improve the accuracy of the algorithm, it is necessary to calibrate the cameras and correct these sources of errors before performing aircraft pose estimation.

## 5. Conclusions

In this article, an aircraft pose estimation method has been developed based on synthetic images of different aircraft and real images captured in the ground laboratory experiment. The proposed method uses line features to describe the structure of the aircraft and exploits the generic geometry structure features of the aircraft to establish line correspondences for pose estimation. A density-based clustering algorithm is used to determine the orientation of the fuselage reference line, while the wing leading edge lines are extracted through vector analysis. Based on our method, a universal framework can be established for aircraft pose estimation without relying on detailed 3D models or other feature datasets. Compared to classical binocular vision methods, no stereo matching process is required in our algorithm and the wide-baseline image pairs are used to obtain accuracy pose estimation results.

As our method can estimate the aircraft pose from image pairs with a large baseline robustly and accurately, our visual system utilizes a wider baseline setup to increase the effective measuring range of binocular vision sensors. And by using our pose estimation method, a universal framework based on vision sensors can be established for aircraft pose estimation without relying on detailed 3D models or other feature datasets.

Our method can also be easily integrated with other algorithms to achieve higher precision pose estimation. For an image sequence recording the aircraft motion, the combination of our algorithm with an extended Kalman filter or particle filter may increase the pose estimation accuracy.

For the aircraft views in which the wings (leading and trailing edges) are visible, the wing leading and trailing edge lines could be detected by modifying the weighted function based on the geometry invariant constraints. Under this condition, if the information about the angle between wing edges and the fuselage (wing patterns) are known, then the perspective-and-line (PnL) method could be used to estimate the aircraft pose and only a monocular camera is required. Developing a more robust structure extraction method and a monocular vision system for aircraft pose estimation will be the focus of our future research.

## Figures and Tables

**Table 1:** The detailed information about the simulation scenarios.

	Model	Camera	Focal Length	Field of View	Image Resolution	Location (x,y,z)
Scene 1	F35	1	800 mm	2.578°×1.934°	1280×960	(0,1000 m,0)
		2	350 mm	5.888°×4.418°	1280×960	(350 m,0,0)
Scene 2	F35	1	800 mm	2.578°×1.934°	1280×960	(0,1000 m,0)
		2	800 mm	2.578°×1.934°	1280×960	(300 m,−900 m,0)
Scene 3	Cessna	1	100 mm	20.408°×15.377°	1280×960	(100 m,0,0)
		2	100 mm	20.408°×15.377°	1280×960	(−200 m,0,0)

**Table 2:** The 3D attitude of F35 model in scene 1.

	1	2	3	4	5	6	7	8	9	10	11	12	13
$\theta_{x}$	0°	0°	0°	0°	0°	0°	0°	0°	0°	0°	0°	−30°	30°
$\theta_{y}$	0°	0°	0°	0°	0°	0°	0°	15°	30°	45°	60°	0°	0°
$\theta_{z}$	0°	30°	60°	90°	−30°	−60°	−90°	0°	0°	0°	0°	0°	0°
